# The Effect of *Bacillus licheniformis*-Fermented Products and Postpartum Dysgalactia Syndrome on Litter Performance Traits, Milk Composition, and Fecal Microbiota in Sows

**DOI:** 10.3390/ani10112044

**Published:** 2020-11-05

**Authors:** Yu-Hsiang Yu, Ting-Yu Hsu, Wei-Jung Chen, Yi-Bing Horng, Yeong-Hsiang Cheng

**Affiliations:** Department of Biotechnology and Animal Science, National Ilan University, Yilan 260, Taiwan; yuyh@niu.edu.tw (Y.-H.Y.); tingyu19961127@gmail.com (T.-Y.H.); wjchen@niu.edu.tw (W.-J.C.); j9551792000@msn.com (Y.-B.H.)

**Keywords:** *Bacillus licheniformis*, fermented product, microbiota, postpartum dysgalactia syndrome, sow

## Abstract

**Simple Summary:**

Supplementation of probiotics can shape the gut microbiota of sows and further influence their offspring’s gut microbiota. Postpartum dysgalactia syndrome (PDS) is a common disease in sows worldwide. Sows with PDS have depressed milk production and increased piglet mortality. The bacterial pathogen is an important factor in the etiology of PDS. *Bacillus licheniformis*-fermented products (BLFP) containing probiotics and antimicrobial substances can prevent disease and improve growth performance in broilers and weaning piglets. However, little is known about the effect of BLFP, PDS, and interaction on litter performance traits, milk composition, and fecal microbiota in sows. In this study, the effects of BLFP and PDS on sows were evaluated. Results show that BLFP supplementation in the diet of sows improves the piglet body weight at weaning. Dietary supplementation of BLFP or PDS differentially regulates the fecal microbiota of sows.

**Abstract:**

This study was designed to evaluate the effects of *Bacillus licheniformis*-fermented products (BLFP) and postpartum dysgalactia syndrome (PDS) on litter performance traits, milk composition, and fecal microbiota in sows in a commercial farrow to finish pig farm. Fifty multiparous cross-bred pregnant sows were randomly assigned to two groups in a completely randomized design. The dietary treatments comprised a basal diet (pregnancy and nursery diet) as control and basal diet supplemented with 1.5 g/kg of BLFP. Sows with PDS in the two groups were further verified 12 h post-partum. Results show that the piglet body weight at weaning was increased in sows fed the BLFP compared to those fed the control diet. The milk fat content of prepartum sows was reduced in sows fed the BLFP. Postpartum sows with PDS had increased milk solid content compared with healthy sows. Microbial composition and species relative abundance analysis indicated distinct bacterial clusters between the groups. The abundance of the family Prevotellaceae in the feces decreased in sows with PDS. BLFP increased the average abundance of the genus *(Eubacterium) coprostanoligenes group* in feces of sows. These findings demonstrate that BLFP in the diet of sows can improve the piglet body weight at weaning and modulate the fecal microbiota of sows. PDS also has an impact on milk composition and fecal microbiota in sows.

## 1. Introduction

Gut microbiota can be shaped by dietary probiotics, thereby improving nutrient utilization, and exerting immunomodulatory effects [1]. Probiotic supplementation is able to modify the fecal microbiota of sows and further affect the gut microbiota in their offspring [2,3]. Probiotics in combination with antibiotic supplementation in the diet of sows and piglets improve growth performance and reduce diarrhea incidence of piglets during the nursery phase [2]. The combined use of *Bacillus licheniformis* and *Bacillus subtilis* in the diet of sows increases feed intake and decreases weight loss during the suckling period [4]. Furthermore, *Bacillus* species-based probiotic supplementation in the diet of sows reduces the diarrhea score and pre-weaning mortality of piglets, leading to an increased number of weaned piglets per litter and body weight at weaning [4]. The fat and protein content in the milk of sows 14 h postpartum is higher in *Bacillus* species-based probiotics-treated group compared with the control group [4].

Postpartum dysgalactia syndrome (PDS) is a multifactor disease in sows with recently reported prevalence varying from 6.0 to 48.2% [5,6]. Sows with PDS exhibit clinical signs of mastitis, dysgalactia, and a fever above 39.5 °C within 12 to 48 h postpartum. In addition to sows, the increased mortality rate and growth retardation are also observed in her offspring due to dysgalactia [7]. PDS leads to enormous economic losses in the swine industry worldwide [8]. Pathogens can invade the mammary gland of sows and cause a local inflammation via the endogenous or the galactogenous route, thereby inducing PDS [7]. Therefore, alteration of gut microbiota of sows may prevent PDS by inhibiting pathogen growth and protecting the mammary glands against fecal contamination.

*B. licheniformis*, an endospore-forming probiotic strain exhibits antibacterial activity in vitro through the producing antibacterial cyclic lipopeptide [9,10]. The surfactin is a *B. licheniformis*-derived antibacterial cyclic lipopeptide [11]. *B. licheniformis*-fermented products (BLFP) containing *B. licheniformis* and surfactin prevent disease and improve growth performance in broilers [12,13]. Recently, it has been demonstrated that BLFP has beneficial effects on the alteration of gut bacterial community and prevention of post-weaning diarrhea in piglets [14,15]. However, to the best of our knowledge, little is known about whether BLFP has a beneficial effect on the sows with PDS and whether this effect can further affect her offspring.

In practice, PDS is treated generally with anti-inflammatory drugs, hormones, or antibiotics. An alternative strategy for PDS control is still rarely investigated. There is an urgent need to demonstrate the positive effects of BLFP for the prevention of PDS in sows before practical application. Therefore, this study aimed to evaluate the effect of BLFP, PDS, and interaction on litter performance traits, milk composition, and fecal microbiota in sows. The results provide a theoretical basis for the use of *B. licheniformis*-fermented products in sows for improving productivity.

## 2. Materials and Methods

### 2.1. Animals and Experimental Design

All animal handling procedures were in compliance with the Institutional Animal Care and Use Committee at the National Ilan University and approved by Institutional Animal Care and Use Committee of National Ilan University (approval number 108-10). Details of the BLFP production are described in our previous study [12]. The concentration of *B. licheniformis* and *B. licheniformis*-derived antibacterial cyclic lipopeptide (surfactin) in BLFP were 4.6 × 10^8^ colony forming unit (CFU)/g and 3 mg/g, respectively. The study was performed from July 2019 to August 2019 on a commercial farrow to finish pig farm in Taiwan. They were managed in a three-week rhythm with a 28 day lactation period. Fifty multiparous cross-bred pregnant sows (Landrace × Yorkshire, parity range 2 to 7) were randomly allocated into two groups in a completely randomized design. The dietary treatments comprised a basal diet (pregnancy and nursery diet) as control and basal diet supplemented with 1.5 g/kg of BLFP (6.9 × 10^5^ CFU/kg of feed). BLFP were formulated by replacing soybean meal from the basal diet. BLFP were fed to the sows 20 d prepartum and 28 d post-partum. A creep feed was used for feeding piglets at day 10 after birth. The diets were formulated to meet or exceed the estimated nutrient requirements for sows recommended by National Research Council (Nutrient Requirements for Swine, 2012, Table 1). The sows were housed in individual pens (180 × 240 cm) with farrowing crates (55 × 185 cm). The farrowing pens were fully slatted with plastic and steel slats. The piglets were housed in the piglet nest of the farrowing pen with a heating lamp set at 30 °C. The sows were fed two times per day with a pellet form of feed. The sows were fed only 1.2 kg per time during gestation. During lactation, the sows were gradually given more feed (range from 1.2 to 3 kg per time) for the first 5 days post-farrowing, followed by ad libitum feeding until weaning. Water was provided ad libitum during the entire experimental period. The cross-fostering among the same treatment group was performed within 24 h after birth. According to a previous study [16], the PDS was determined if at least two of the following clinical criteria were fulfilled: 1. rectal temperature higher than 39.5 °C 12 h post-partum, 2. anorexia, and 3. mastitis. The rectal temperature of the sows was measured two times and then the average was calculated. Finally, the number of healthy sows fed only a basal diet, healthy sows fed BLFP, sows with PDS fed only a basal diet, and sows with PDS fed BLFP were 12, 17, 10, and 11, respectively. The average parity of healthy sows fed only a basal diet, healthy sows fed BLFP, sows with PDS fed only a basal diet, and sows with PDS fed BLFP was 3.3, 3.6, 3.2, and 3.5, respectively. The farm had a previous history of pre-weaning diarrhea syndrome caused by *Porcine epidemic diarrhea virus* (PEDV) as evidenced by pathological and microbiological examinations. Breeding animals were vaccinated against *Parvovirus*, *Japanese encephalitis virus*, *Erysipelothrix rhusiopathiae*, *Bordetella bronchiseptica*, *Pasteurella multocida*, *Aphthovirus, Pseudorabies virus*, and *Pestivirus classical swine fever virus*. New-born piglets were also vaccinated against *Pestivirus classical swine fever virus*.

### 2.2. Colostrum Analysis

Colostrum was collected 12 h prepartum and postpartum from the first, third, and sixth teats of the sow for a total amount of 15 mL following administration of oxytocin. The sows were randomly chosen, and colostrum was collected from least three individual sows per group at the same time. Next, the samples were transported to the Laboratory of Milk Assessment and Analysis of the Livestock Research Institute (Hsinchu, Taiwan) to determine the basic composition of milk. Colostrum was analyzed for fat, protein, lactose, solids-not-fat, and solids on the CombiFoss 7 analyzer (FOSS Electric, Hillerød, Denmark).

### 2.3. 16S Ribosomal RNA Sequencing and Analysis

Feces from individual sows were freshly collected 12 h postpartum by rectal grab. The sows were randomly chosen, and feces were collected from least three individual sows per group at the same time. Details of the 16S ribosomal RNA sequencing and analysis are provided in a previous study [14]. Briefly, total genomic DNA from fecal content was purified, quantified, and amplified. PCR products of 16S rRNA genes were purified for sequencing library construction. The paired-end sequencing libraries (2 × 300 bp) were generated. The obtained sequences were clustered into bins called operational taxonomic units (OTUs) based upon 97% similarity and sequencing read data were aligned with the Genomes Online Database. A Venn diagram, taxonomic assignment, and alpha diversity analysis were analyzed. A heatmap analysis was performed based on the species composition and relative abundance of each sample. The microbial composition, including principal component analysis (PCA), principal coordinate analysis (PCoA) plots for both unweighted (qualitative traits) and weighted UniFrac distance metric (quantitative traits), and plots of beta-diversity distances between communities for both unweighted and weighted UniFrac distance metric were calculated.

### 2.4. Statistical Analysis

Two-group comparison (C and F) was analyzed using Student’s *t*-test (two-tailed). Differences between treatments were examined with two-way ANOVA using SAS software (version 9.4, 2012; SAS Institute, Cary, NC, USA). The experimental unit was the sow and her litter, and the fixed effects included in the model were diet (C and F), status (health and PDS), and its interaction. The Tukey’s honestly significant difference test was used to test differences among sample means for significance. A *p* value of less than 0.05 was statistically significant, and a *p* value between 0.05 and 0.1 was considered a trend.

## 3. Results

### 3.1. The Litter Performance Traits and Milk Composition of Sows in Response to Bacillus licheniformis-Fermented Product Supplementation

The effect of BLFP on litter performance traits is shown in Table 2. Results show that there were no statistically significant differences in the parameters of litter performance traits (number of piglets totally born per litter, number of piglets born alive per litter, number of weaned piglets per litter, number of dead piglets during suckling period per litter, pre-weaning mortality per litter, and piglet body weight at birth per litter) except piglet body weight at weaning per litter. The piglet body weight at weaning was increased (*p* < 0.05) in sows fed the BLFP compared to those fed the control diet. Next, the interactions between diet (with or without BLFP) and status (health or PDS) in sows were investigated. As expected, sows with PDS exhibited higher rectal temperature compared to healthy sows (*p* < 0.05) (Table 3). Diet had a significant effect on piglet body weight at weaning (*p* < 0.05) (Table 3). The average feed intake of healthy sows fed only a basal diet, healthy sows fed BLFP, sows with PDS fed only a basal diet, and sows with PDS fed BLFP was 6.40 kg, 6.13 kg, 6.18 kg, and 5.99 kg, respectively. No significant difference was observed in the feed intake among the groups during lactation. The effect of BLFP on milk composition is shown in Table 4. Diet had a significant effect on the fat content of prepartum sows (*p* < 0.05), whereas this effect was not observed in the fat content of postpartum sows. The fat content of prepartum sows was lower in the group treated with BLFP (*p* < 0.05). There was a trend for interaction between diet and status on the protein content (*p* = 0.097) and solid content (*p* = 0.051) of prepartum sows. A trend of interaction between diet and status was observed in the protein content (*p* = 0.066), solids-not-fat content (*p* = 0.075), and solid content (*p* = 0.098) of postpartum sows. Status had a significant effect on the solid content of postpartum sows (*p* < 0.05). Postpartum sows with PDS had increased solid content compared with healthy sows (*p* < 0.05).

### 3.2. Effect of Bacillus licheniformis-Fermented Products and Postpartum Dysgalactia Syndrome on the Fecal Microbiota of Sows

**Figure 1:**
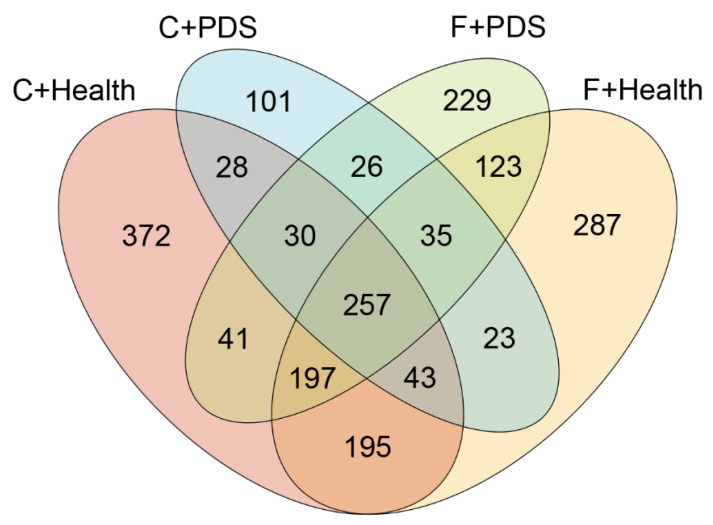
Operational taxonomic unit distribution across the analyzed fecal content. Venn diagram representing the number of unique and shared operational taxonomic units among the four groups: healthy sows plus control diet group (C + Health, *n* = 7), sows with postpartum dysgalactia syndrome (PDS) plus control diet group (C + PDS, *n* = 3), healthy sows plus *B. licheniformis*-fermented product group (F + Health, *n* = 6), and sows with PDS plus *B. licheniformis*-fermented product group (F + PDS, *n* = 4).

**Figure 2:**
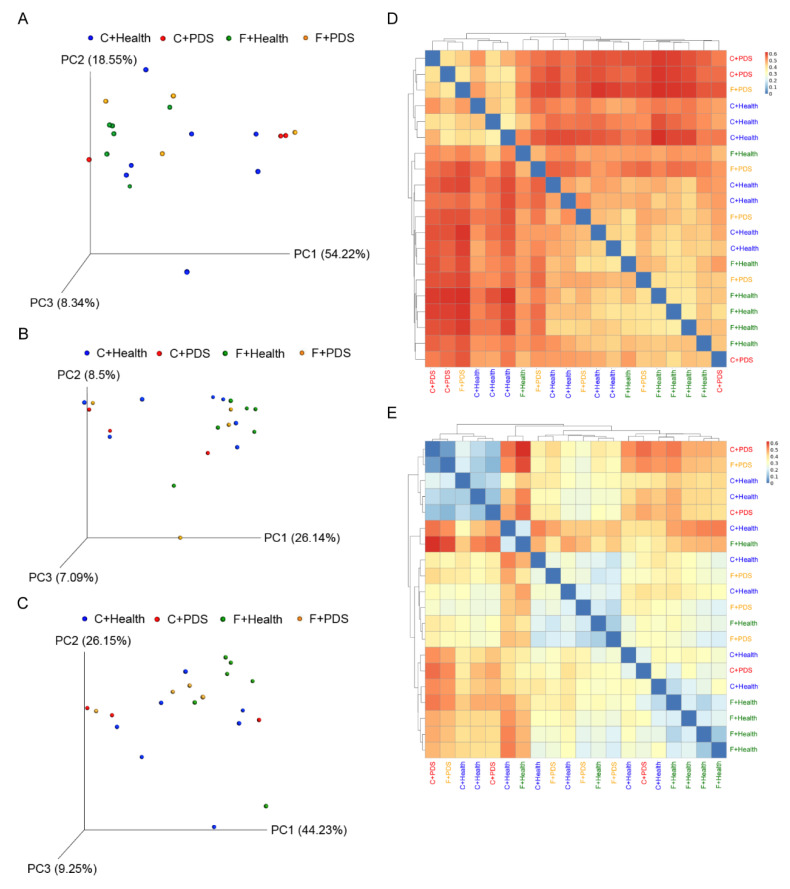
Microbial composition analysis of fecal content. (**A**) Principal component analysis plot of healthy sows plus control diet group (C + Health, *n* = 7), sows with PDS plus control diet group (C + PDS, *n* = 3), healthy sows plus *B. licheniformis*-fermented product group (F + Health, *n* = 6), and sows with PDS plus *B. licheniformis*-fermented product group (F + PDS, *n* = 4). Principal coordinate analysis plots of qualitative traits (unweighted UniFrac distance metric) (**B**) and quantitative traits (weighted UniFrac distance metric) (**C**) of the fecal microbial composition from C + Health (*n* = 7), C + PDS (*n* = 3), F + Health (*n* = 6), and F + PDS (*n* = 4). (**D**) Beta diversity analysis plot of the fecal content from C + Health (*n* = 7), C + PDS (*n* = 3), F + Health (*n* = 6), and F + PDS (*n* = 4) based on unweighted UniFrac distance metric. (**E**) Beta diversity analysis plot of the fecal content from C + Health (*n* = 7), C + PDS (*n* = 3), F + Health (*n* = 6), and F + PDS (*n* = 4) based on weighted UniFrac distance metric.

The averages of high-quality reads and operational taxonomic units (OTUs) from the fecal content of sows were 23388.6 and 9178.0 after stringent quality trimming of raw data. Diet and status did not affect the fecal species richness (Chao1 and Fisher alpha estimator) (Table 5). There was a tendency of increasing fecal species evenness (Shannon) in response to BLFP supplementation (*p* = 0.085). The Venn diagram showed an area of overlap (257 OTUs, core) that was shared by four of the plotted groups (Figure 1). Furthermore, 372, 101, 229, and 287 unique OTUs were found in the healthy sows plus control diet group, sows with PDS plus control diet group, sows with PDS plus BLFP group, and healthy sows plus BLFP, respectively. Additionally, 28 OTUs were detected in both the healthy sows plus control diet group and sows with PDS plus control diet group; 26 OTUs were found in both the sows with PDS plus control diet group and sows with PDS plus BLFP group; 123 OTUs were found in both the sows with PDS plus BLFP group and healthy sows plus BLFP group; 195 OTUs were found in both the healthy sows plus BLFP group and healthy sows plus control diet group. The PCA plot revealed statistically significant discrimination in the fecal microbiota among the groups (PC1, 54.22%; PC2, 18.55%; PC3, 8.34%; Figure 2A). The PCoA of qualitative traits (unweighted UniFrac distances) indicated that fecal microbiota was clearly separated among the groups (PC1, 26.14%; PC2, 8.5%; PC3, 7.09%; Figure 2B). The PCoA of quantitative traits (weighted UniFrac distances) also indicated a clear pattern of separation among the groups (PC1, 44.23%; PC2, 26.15%; PC3, 9.25%; Figure 2C). Plots of beta diversity analysis based on qualitative traits and quantitative traits also showed a clear separation in the fecal microbiota among the groups (Figure 2D,E).

### 3.3. Effects of Bacillus licheniformis-Fermented Products and Postpartum Dysgalactia Syndrome on the Fecal Bacterial Taxonomic Composition of Sows

**Figure 3:**
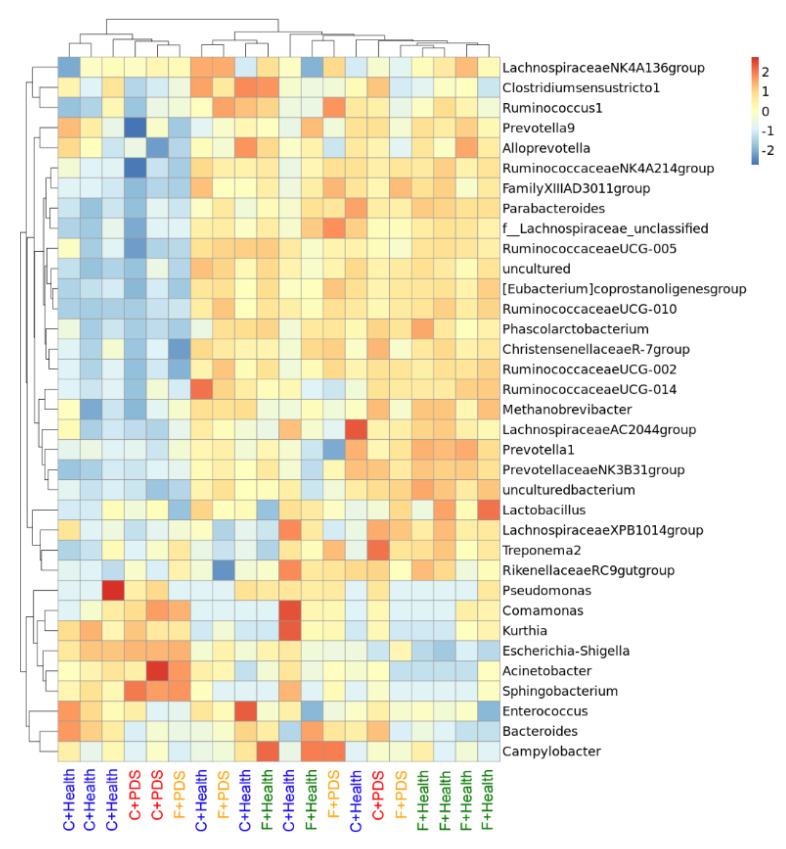
Species abundance heat map showing normalized values of 35 differentially abundant genera of four groups. Group and genus names are plotted on the *X*-axis and *Y*-axis, respectively. Red color indicates that the Z-score of a specific genus is larger than the mean, whereas the blue color indicates the opposite.

Based on the genus-level heatmap, two distinct clusters were separated between the healthy sows plus control diet (left side) and healthy sows plus BLFP group (right side) (Figure 3). The results of bacterial classification in the fecal content of sows are shown in Table 6. At the phylum level, the diet tended to regulate the abundance of the phyla Firmicutes (*p* = 0.083) and Proteobacteria (*p* = 0.072) in the feces. An increasing trend in the abundance of the phylum Firmicutes and a decreasing trend in the abundance of the phylum Proteobacteria in response to BLFP supplementation were observed. Status tended to modulate the abundance of the phyla Proteobacteria (*p* = 0.086) and Bacteroidetes (*p* = 0.078) in the feces. An increasing trend in the abundance of the phylum Proteobacteria and a decreasing trend in the abundance of the phylum Bacteroidetes in the feces of sows with PDS were observed. At the class level, the diet tended to regulate the abundance of the classes Clostridia (*p* = 0.060) and Gammaproteobacteria (*p* = 0.076) in the feces. Status tended to modulate the abundance of the classes Gammaproteobacteria (*p* = 0.090) and Bacteroidia (*p* = 0.078) in the feces. At the order level, the diet tended to regulate the abundance of the order Clostridiales (*p* = 0.060) in the feces. Status tended to modulate the abundance of the orders Enterobacteriales (*p* = 0.057) and Bacteroidales (*p* = 0.061) in the feces. At the family level, the diet tended to regulate the abundance of the family Ruminococcaceae (*p* = 0.053) in the feces. Status tended to modulate the abundance of the family Enterobacteriaceae (*p* = 0.057) in the feces. Diet had a significant effect on the abundance of the family Prevotellaceae (*p* < 0.05) in the feces. The abundance of the family Prevotellaceae in the feces was decreased in sows with PDS (*p* < 0.05). At the genus level, status tended to regulate the abundance of the genera *Escherichia-Shigella* (*p* = 0.057), *Prevotella 9* (*p* = 0.059), and *Ruminococcaceae UCG-005* (*p* = 0.072) in the feces. Diet had a significant effect on the abundance of the genus *(Eubacterium) coprostanoligenes group* (*p* < 0.05) in the feces. The abundance of the genus *(Eubacterium) coprostanoligenes group* in the feces was increased in response to BLFP supplementation (*p* < 0.05).

## 4. Discussion

Here, we demonstrated for the first time that BLFP supplementation in the diet of sows improved the piglet body weight at weaning. We confirmed that BLFP supplementation in the diet of sows decreased the milk fat content of prepartum sows and PDS increased the milk solid content of postpartum sows. Principal component analysis, principal coordinates analysis, and a species abundance heat map in the feces of sows revealed distinct bacterial clusters between the groups. The abundance of the family Prevotellaceae was lower in the feces in sows with PDS. The abundance of the genus *(Eubacterium) coprostanoligenes group* was higher in the feces in the group treated with BLFP.

Previous studies have demonstrated that the use of probiotics in the diet of sows can improve the litter performance and milk yield [4,17,18]. Dietary *B. subtilis* supplementation in the diet of sows has been shown to increase the piglet body weight at birth per litter and piglet body weight at weaning [19,20]. However, a recent study has shown that dietary supplementation of *B. subtilis* in the diet of sows and their neonates did not improve the performance [21]. The administration of *B. mesentericus* in combination with *Clostridium butyricum* and *Enterococcus faecalis* in the diet of sows and their neonates can reduce post-weaning diarrhea and improve growth performance [22]. The combined use of *B. subtilis* and *B. licheniformis* in sows decreases the pre-weaning mortality per litter and increases the number of weaned piglets per litter and piglet body weight at weaning [4]. In addition to probiotic supplementation, the beneficial effects of fermented feed on the reproductive and lactation performance of sows and the growth performance of piglets has been observed [23,24]. Our previous studies have confirmed that BLFP exhibits antibacterial activity and prevents post-weaning diarrhea in weaning piglets [10,14,15]. Here, we further demonstrated that dietary supplementation of BLFP in the diet of sows during gestation and lactation can improve the piglet body weight at weaning. The milk fat content in prepartum (5.9% for health and 7.28% for PDS) or postpartum (6.44% for health and 7.73% for PDS) sows of the control group was higher than prepartum (4.66% for health and 4.07% for PDS) or postpartum (5.18% for health and 6.38% for PDS) sows of the BLFP-treated group. The result indicates that BLFP supplementation negatively affects the milk fat content of sows. Although it did not reach statistical significance, the average milk protein content (18.46%) of prepartum BLFP-fed healthy sows was higher than in other groups. However, the average milk protein content (11.23%) of postpartum BLFP-fed healthy sows was not the highest among the groups. Based on current findings, it is hard to conclude that milk composition plays a role in improving the piglet body weight at weaning in the BLFP-treated group. According to a previous study [24], the fermented feed can increase milk yield and milk immunoglobulin Acontent of sows, leading to the improved growth performance of piglets. Whether supplementing sow diets with BLFP can increase milk yield and milk immunoglobulin A content needs to be elucidated in the future. Collectedly, the findings indicate that dietary supplementation of BLFP in the diet of sows has beneficial effects on the piglet body weight at weaning. The detailed mechanisms of BLFP in improving litter performance of sows remain to be investigated.

It has been reported that probiotic supplementation is able to modify the fecal microbiota of sows and further shape the gut microbiota in their offspring [2,3]. Dietary *B. subtilis* supplementation can elevate the abundance of the *Lactobacillus* species in the colon of sows and reduce the abundance of *Clostridium perfringens* and *Escherichia coli* [3,19]. In contrast, the fecal microbial population of sows is not changed in response to administration of *B. mesentericus* in combination with *Clostridium butyricum* and *Enterococcus faecalis*, whereas the abundance of genus *Bifidobacterium* in the ileum of their offspring is increased [22]. Dietary supplementation of *B. subtilis* in the diet of sows increases the abundance of *Bacillus* species in the feces [21]. Previous studies have mainly used traditional methods for investigating the effects of probiotic supplementation on the microbiota of sows and their offspring. Here, we found that BLFP-fed sows tended to have increased bacterial species evenness in the feces and modified bacterial composition. Dietary supplementation of BLFP in the diet of sows also increased the genus *(Eubacterium) coprostanoligenes group* abundances in the feces. The genus *(Eubacterium) coprostanoligenes group*, a cholesterol-reducing anaerobe, catalyzes the conversion of cholesterol to coprostanol [25]. It has been reported that dietary *B. subtilis* supplementation increases the genus *(Eubacterium) coprostanoligenes group* abundances in the feces of weaning piglets [26]. The abundance of the genus *(Eubacterium) coprostanoligenes group* in the feces is negatively correlated with the diarrhea incidence of piglets [27]. Taken together, supplementation of BLFP in the diet of sows can modify the gut microbiota and probably increase the beneficial bacterial population, thereby reducing the load of pathogens in the environment. Whether feeding the BLFP to sows also has an impact on the gut microbiota of their offspring still needs to be investigated.

PDS is a major disease occurring in sows and many pathogens can be isolated from the milk and feces of sows with PDS [7]. The coliform bacteria, such as genus *Escherichia*, *Citrobacter*, *Enterobacter*, and *Klebsiella*, are frequently found in sows with PDS [7]. It has been reported that dietary fermented feed supplementation can reduce the pathogen challenge of the neonatal environment [23]. In the present study, we found that sows with PDS tended to have increased abundance of the genus *Escherichia-Shigella* and decreased abundance of the genera *Prevotella 9* and *Ruminococcaceae UCG-005* in the feces. The genera *Escherichia-Shigella* and *Prevotella 9* are classified as pathogenic bacteria and beneficial bacteria in pigs, respectively [14,15,27]. Furthermore, postpartum sows with PDS had increased solid content compared with healthy sows. However, PDS did not have an impact on the piglet body weight at weaning. A previous study has shown that the combined use of *B. subtilis* and *B. licheniformis* in sows tends to reduce the incidence of PDS and the return to oestrus interval [4]. In the present study, no significant interaction between BLFP and PDS on the improvement in litter performance traits, milk composition, and fecal microbiota was found. These observations suggest that sows with PDS can cause gut microbiota imbalance. Whether these changes of fecal microbiota have a direct effect on the incidence of PDS in sows remains to be confirmed.

Since the farm had a previous history of pre-weaning diarrhea syndrome caused by PEDV, the PEDV may be a major reason for the increased pre-weaning mortality in the present study. However, the PEDV infection rate, recovery rate, and died from porcine epidemic diarrhea rate in piglets is unclear. Based on the current findings, the average pre-weaning mortality per litter of the control group was 38.37% in this PEDV-positive farm. In the BLFP group, the average pre-weaning mortality per litter was not improved (40.68%). This result implies that PEDV may be prevalent and has a similar infection rate in these two groups. Further, BLFP supplementation in the diet of sows cannot alleviate the pre-weaning mortality per litter. Whether there is any interaction between BLPF, PDS, and PEDV on litter performance traits still needs to be investigated.

## 5. Conclusions

The field study confirmed that BLFP supplementation in the diet of sows during gestation and lactation can improve the piglet body weight at weaning. BLFP supplementation in the diet of sows and PDS differentially regulates the fecal microbiota. These findings are particularly important for providing field trial evidence of how BLFP can be applied in the swine industry for microbiota manipulation of sows to improve piglet body weight at weaning.

## Figures and Tables

**Table 1:** Basal diet composition and nutritional value.

Items	Diet	
	Pregnancy	Nursery
Ingredient, g kg^−1^		
Corn	500	680
Wheat bran	333	0
Soybean meal, 48% CP	130	280
Pregnancy vitamin and mineral, premix ^1^	4	0
Nursery vitamin and mineral, premix ^2^	0	4
Fermented products	0	0
Chemical composition of the diets, g kg^−1^		
Crude protein	151.8	183.9
Crude fiber	48.1	31.8
Analyzed calcium	10.0	9.8
Analyzed total phosphorus	6.0	5.2
Lysine	5.7	8.5
Methionine + Cystine	4.2	5.1
Threonine	4.0	5.4
Tryptophan	1.5	1.9
ME, kcal/kg	2886	3300

^1^ Supplied per kg of diet: vitamin A, 112,500 IU; vitamin D3, 20,000 IU; vitamin E, 500 IU; vitamin K3, 25 mg; vitamin B1, 25 mg; vitamin B2, 87.5 mg; pantothenic acid, 250 mg; vitamin B6, 31 mg; niacin, 450 mg; folic acid, 30 mg; biotin, 2.25 mg; choline, 8170 mg; vitamin B12, 400 μg; Ca, 170 g; P, 17 g; Na, 50 g; Mn, 625 mg; Zn, 1375 mg; Fe, 1000 mg; Cu, 1875 mg; I, 23 mg; Se, 7.5 mg. ^2^ Supplied per kg of diet: vitamin A, 112,500 IU; vitamin D3, 20,000 IU; vitamin E, 500 IU; vitamin K3, 25 mg; vitamin B1, 25 mg; vitamin B2, 87 mg; pantothenic acid, 250 mg; vitamin B6, 31 mg; niacin, 450 mg; folic acid, 30 mg; biotin, 2.5 mg; choline, 5000 mg; vitamin B12, 400 μg; Ca, 210 g; P, 42.5 g; Na, 51 g; Mn, 625 mg; Zn, 1375 mg; Fe, 1000 mg; Cu, 175 mg; I, 23 mg; Se, 7.5 mg.

**Table 2:** The litter performance traits of sows in response to *Bacillus licheniformis*-fermented product supplementation.

Item	C ^1^	F ^2^	SEM ^3^	*p*-Value ^4^
Number of piglets totally born/L	12.47 ^5^	13.48	0.60	0.408
Number of piglets born alive/L	10.65	11.91	0.57	0.274
Number of weaned piglets/L	9.00	8.37	0.41	0.478
Number of dead piglets during suckling period/L	5.20	6.33	0.66	0.378
Pre-weaning mortality (%)/L	38.37	40.68	4.05	0.784
Piglet body weight (kg) at birth/L	1.55	1.47	0.04	0.338
Piglet body weight (kg) at weaning/L	6.61	7.22	0.15	0.041

^1^ C = pregnancy and nursery diet. ^2^ F = pregnancy and nursery diet supplemented with BLFP. ^3^ SEM = standard error of mean. ^4^ Analyzed using Student’s *t*-test (two-tailed). ^5^ Data are mean values of 12–14 L per group.

**Table 3:** Effect of *Bacillus licheniformis*-fermented products and postpartum dysgalactia syndrome on the rectal temperature, piglet body weight at weaning of sows, and feed intake.

	C ^1^		F ^2^			*p*-Value		
Item	Health	PDS ^4^	Health	PDS	SEM ^3^	Diet	Status	Diet × Status
Rectal temperature of sows (°C)	39.02 ^5^	39.88	38.96	40.07	0.088	0.625	<0.001	0.327
Piglet body weight at weaning (kg)	6.92 ^6^	6.23	7.24	7.19	0.147	0.028	0.202	0.265
Feed intake of sows (kg/d/head)	6.40	6.18	6.12	5.99	0.119	0.387	0.507	0.888

^1^ C = pregnancy and nursery diet. ^2^ F = pregnancy and nursery diet supplemented with *Bacillus licheniformis*-fermented products (BLFP). ^3^ SEM = standard error of mean. ^4^ PDS = postpartum dysgalactia syndrome. ^5^ Data are mean values of 4–18 sows per group. ^6^ Data are mean values of 10–14 L per group.

**Table 4:** Effect of *Bacillus licheniformis*-fermented products and postpartum dysgalactia syndrome on the milk composition of sows.

Items	C ^1^		F ^2^			*p*-Value		
	Health	PDS ^4^	Health	PDS	SEM ^3^	Diet	Status	Diet × Status
Prepartum								
Fat (%)	5.90 ^5^	7.28	4.66	4.07	0.358	0.004	0.575	0.168
Protein (%)	16.69	17.24	18.46	16.32	0.373	0.595	0.320	0.097
Lactose (%)	2.09	1.94	2.00	2.18	0.075	0.664	0.909	0.346
SNF (%)	20.88	21.28	22.55	20.60	0.347	0.501	0.290	0.117
Solid (%)	26.78	28.57	27.20	24.65	0.500	0.110	0.719	0.051
Postpartum								
Fat (%)	6.44	7.73	5.18	6.38	0.416	0.167	0.186	0.959
Protein (%)	9.20	12.65	11.23	10.61	0.497	0.998	0.193	0.066
Lactose (%)	3.37	2.84	2.94	2.81	0.125	0.410	0.234	0.463
SNF (%)	14.66	17.59	16.26	15.52	0.454	0.815	0.280	0.075
Solid (%)	21.10	25.33	21.44	21.90	0.521	0.171	0.042	0.098

^1^ C = pregnancy and nursery diet. ^2^ F = pregnancy and nursery diet supplemented with BLFP. ^3^ SEM = standard error of mean. ^4^ PDS = postpartum dysgalactia syndrome. ^5^ Data are mean values of 3–7 sows per group.

**Table 5:** Effect of *Bacillus licheniformis*-fermented products and postpartum dysgalactia syndrome on the microbial diversity in the fecal content of sows.

Item	C ^1^		F ^2^			*p*-Value		
	Health	PDS ^4^	Health	PDS	SEM ^3^	Diet	Status	Diet × Status
Chao ^1^	319.14 ^5^	241.67	426.67	337.25	30.193	0.117	0.192	0.924
Fisher alpha	5.31	4.66	6.66	4.47	0.426	0.515	0.122	0.391
Shannon	3.61	2.93	4.65	3.69	0.251	0.085	0.111	0.779
Enspie	44.23	31.66	60.57	47.04	4.68	0.114	0.188	0.960

^1^ C = pregnancy and nursery diet. ^2^ F = pregnancy and nursery diet supplemented with BLFP. ^3^ SEM = standard error of mean. ^4^ PDS = postpartum dysgalactia syndrome. ^5^ Data are mean values of 3–7 sows per group.

**Table 6:** Effect of *Bacillus licheniformis*-fermented products and postpartum dysgalactia syndrome on the bacterial classification within the fecal content of sows.

Items	Relative Abundance (%)					*p*-Value		
	C ^1^		F ^2^					
	Health	PDS ^4^	Health	PDS	SEM ^3^	Diet	Status	Diet × Status
Phylum								
*Firmicutes*	55.63 ^5^	49.26	62.75	65.16	2.976	0.083	0.755	0.490
*Proteobacteria*	21.42	32.20	5.51	20.74	3.724	0.072	0.086	0.758
*Bacteroidetes*	20.97	14.92	26.12	10.04	2.886	0.983	0.078	0.405
Class								
*Clostridia*	52.33	48.66	60.20	64.30	2.828	0.060	0.971	0.512
*Gammaproteobacteria*	21.18	31.58	5.11	20.56	3.736	0.076	0.090	0.729
*Bacteroidia*	20.97	14.92	26.12	10.04	2.886	0.983	0.078	0.405
*Erysipelotrichia*	0.59	0.018	0.31	0.22	0.119	0.886	0.208	0.353
*Methanobacteria*	0.90	1.33	2.16	0.86	0.305	0.543	0.503	0.197
*Bacilli*	2.41	0.39	1.66	0.31	0.472	0.680	0.105	0.735
Order								
*Clostridiales*	52.33	48.66	60.20	64.30	2.828	0.060	0.970	0.512
*Enterobacteriales*	18.10	29.05	4.96	20.00	3.334	0.100	0.057	0.752
*Bacteroidales*	20.84	12.99	26.08	9.62	2.985	0.879	0.061	0.485
*Erysipelotrichales*	0.59	0.018	0.31	0.22	0.120	0.886	0.208	0.353
*Methanobacteriales*	0.90	1.33	2.16	0.86	0.305	0.543	0.503	0.197
*Lactobacillales*	1.42	0.17	1.64	0.27	0.397	0.855	0.143	0.941
Family								
*Lachnospiraceae*	40.56	41.74	43.37	48.46	1.459	0.131	0.310	0.523
*Ruminococcaceae*	7.46	2.99	11.41	11.89	1.514	0.053	0.525	0.433
*Enterobacteriaceae*	18.10	29.05	4.96	20.00	3.334	0.100	0.057	0.752
*Christensenellaceae*	1.46	2.84	3.45	2.53	0.502	0.444	0.831	0.298
*Clostridiaceae 1*	2.33	0.96	1.23	0.59	0.495	0.504	0.368	0.741
*Prevotellaceae*	12.12	8.26	18.43	6.25	1.840	0.545	0.035	0.249
*Erysipelotrichaceae*	0.59	0.02	0.31	0.22	0.119	0.886	0.208	0.353
*Methanobacteriaceae*	0.90	1.33	2.16	0.86	0.305	0.543	0.503	0.197
*Lactobacillaceae*	0.17	0.04	1.36	0.21	0.305	0.303	0.331	0.434
Genus								
*Lachnospiraceae NK4A136 group*	37.50	40.52	39.98	45.74	1.48	0.223	0.168	0.657
*Ruminococcaceae UCG-014*	2.18	0.54	1.36	0.90	0.66	0.878	0.488	0.693
*Escherichia-Shigella*	18.05	29.02	4.94	20.00	3.33	0.100	0.057	0.753
*Clostridium sensu stricto 1*	2.32	0.94	1.19	0.49	0.49	0.470	0.346	0.755
*Christensenellaceae R-7 group*	1.44	2.84	3.45	2.51	0.50	0.443	0.831	0.289
*Prevotella 9*	8.75	5.62	11.57	3.81	1.34	0.851	0.059	0.400
*Ruminococcaceae UCG-005*	1.37	0.32	1.88	1.21	0.23	0.138	0.072	0.677
*Methanobrevibacter*	0.90	1.33	2.16	0.86	0.30	0.543	0.503	0.197
*[Eubacterium] coprostanoligenes group*	0.83	0.36	1.90	2.03	0.26	0.014	0.745	0.553
*Ruminococcaceae UCG-013*	0.26	0.06	0.17	0.21	0.07	0.834	0.610	0.430

^1^ C = pregnancy and nursery diet. ^2^ F = pregnancy and nursery diet supplemented with BLFP. ^3^ SEM = standard error of mean. ^4^ PDS = postpartum dysgalactia syndrome. ^5^ Data are mean values of 3–7 sows per group.
